# Bioinspired Coiled Composite Hydrogel Evaporator with Carbonized Hair for Efficient Solar Desalination and Salt Rejection

**DOI:** 10.1021/acsomega.6c07905

**Published:** 2026-08-18

**Authors:** Yi Liu, Liqiang Zhang, Haoqi Ni, Shuozhen Chen, Ran Li, Jiaxian Zheng, Xiangfeng Lin, Weiwei Zhao, Zhanhui Yuan

**Affiliations:** † College of Materials Engineering, 12449 Fujian Agriculture and Forestry University, Fuzhou 350108, P.R. China; ‡ State Key Laboratory of Advanced Papermaking and Paper-based Materials, South China University of Technology, Guangzhou 510640, P.R. China; § Birmingham Centre for Energy Storage & School of Chemical Engineering, 1724 University of Birmingham, Edgbaston B15 2TT, U.K.

## Abstract

Interfacial solar-driven seawater desalination has attracted significant attention. However, achieving both high evaporation rates and excellent salt resistance through advanced structural design and material optimization remains a considerable challenge. Inspired by coiled structures, this study presents a coil-structured solar evaporator (C-SPCH) fabricated by coiling a composite hydrogel containing carbonized hair, sodium alginate, and PVA (poly­(vinyl alcohol)). Its coiled architecture enables fast capillary channels and interconnected pores for enhanced water transport and salt tolerance. The irregular surface structure notably increases light-harvesting efficiency, while embedded hydrophilic groups smartly control water molecule states, cutting evaporation enthalpy. Therefore, the C-SPCH evaporator exhibits exceptional performance, achieving an evaporation rate of up to 5.36 kg·m^–2^·h^–1^ and an outstanding solar-vapor conversion efficiency of 150% (accounting for the additional environmental energy harvested by the three-dimensional coiled structure). It sustains efficient evaporation across a wide salinity range (0–25 wt %) and operates stably for 10 days in 15 wt % brine without significant performance degradation. This study offers insights into the structural design of solar-driven water evaporation systems, highlighting its significant potential for applications in both seawater desalination and high-salinity wastewater treatment.

## Graphical abstract

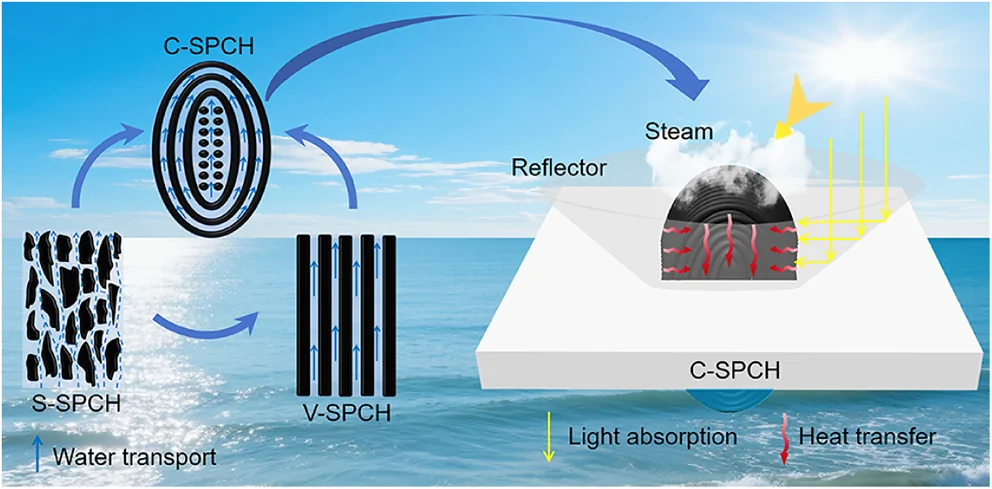

## 1 Introduction

Freshwater scarcity is one of the most critical global challenges today. Currently, 55% of the global population experiences clean water shortages for at least one month per year.^1^ Interfacial solar-driven seawater desalination has garnered widespread attention due to its reliance on abundant solar energy and its inherently sustainable and environmentally friendly approach to steam generation.^2^ For a solar thermal system to work efficiently, the absorbing material should convert sunlight into heat well, the system must keep the heat from escaping, and there must always be enough water available.^3^ Neglecting any of these will lower the efficiency. However, most existing evaporators involve complex manufacturing processes and high production costs. Consequently, researchers have developed novel hydrogel-based evaporators, often characterized by their ease of fabrication and optimized for efficient water transport.^4^ These hydrogel-based materials achieve high evaporation rates by effectively reducing the enthalpy of water evaporation and facilitating the efficient utilization of thermal energy during the process.

Photothermal materials play a crucial role in enhancing the evaporation rate and photothermal conversion efficiency of solar evaporators. The selection and design of these materials primarily involve plasmonic, carbon-based, organic polymer, semiconductor, and biomass-derived materials.^5^ However, for practical applications, low cost and facile fabrication are crucial factors. Conversely, many of these materials often involve complex preparation processes and high production costs. Consequently, biomass materials have garnered extensive attention in photothermal applications due to their low cost, broad spectral absorption, highly porous structures, and low thermal conductivity.^6^ Recently, various biomass materials, including durian peel,^7^ potato slices,^8^ bamboo leaves,^9^ corn straw^10^ and mushrooms,^11^ have been utilized to construct interfacial solar evaporators. Human hair, despite being an abundant organic waste product, is largely discarded as refuse. Building on our previous work, where we developed a 3D solar evaporator based on the principle of blackbody radiation for broadband light absorption and heat conversion,^12^ we now address the challenge of biodegradability.^13^ To enhance the evaporator’s stability and salt-rejection capabilities in seawater, we have incorporated carbonized hair as the photothermal material within a hydrogel matrix. Compared with conventional lignocellulosic biomass precursors such as fruit peels, leaves, stalks, and tubers, human hair is a keratin-rich, proteinaceous waste with intrinsically abundant nitrogen and sulfur-containing amino-acid moieties. This chemistry provides a chemically distinctive carbon precursor without requiring the photothermal function to remain coupled to a preserved native architecture.

However, most previous studies have relied solely on the native structures of biomass materials. While these materials may exhibit some performance as solar evaporators, they are inherently limited by their intrinsic structural imperfections.^14^ Therefore, further structural design and optimization of biomass-derived solar thermal materials remain challenging, yet are crucial for enhancing and precisely controlling solar-thermal performance.^15,16^ Conventional evaporators are typically designed with a two-dimensional planar configuration, leaving the light absorption characteristics and water transport behavior on curved surfaces largely unexplored.^17^

Inspired by coil structures, this study proposes an innovative coil-shaped interfacial solar evaporator (Figure 1a and 1b). Carbonized human hair (C-Hair) serves as the photothermal material, integrated with sodium alginate (SA) and poly­(vinyl alcohol) (PVA) through a wet-spinning process to form a novel composite hydrogel tube, designated as HSPCH (HSP hydrogel incorporated with carbonized hair, C-Hair). This tube is further assembled into a coiled evaporator structure (C-SPCH). Experimental results demonstrate that the C-SPCH evaporator exhibits outstanding performance, achieving a high evaporation rate of 5.36 kg·m^–2^·h^–1^ and an outstanding solar-vapor conversion efficiency of 150%, maintaining high evaporation efficiency across a broad range of salinities (0–25 wt %), and operating continuously for 10 days in 15 wt % brine without significant performance degradation. The excellent performance of the evaporator stems from its unique coil-shaped design. This is because the design fosters the formation of rapid capillary channels between hydrogel fibers, which synergize with the material’s intrinsic pore structures to significantly enhance water transport and salt resistance, as verified by comparative experiments with vertically aligned (V-SPCH) and bulk-structured (S-SPCH) hydrogel evaporators (Figure 1a). Meanwhile, the irregular surface morphology resulting from the coiled architecture improves light-trapping capability, leading to superior light absorption compared to conventional bulk hydrogels. Moreover, the abundant hydrophilic groups within the hydrogel effectively modulate water molecule interactions, considerably reducing the evaporation enthalpy and thereby improving evaporation efficiency. This study offers new insights into the structural design of high-performance solar-driven water evaporation systems, with promising applications in desalination and high-salinity wastewater treatment.

**Figure 1:**
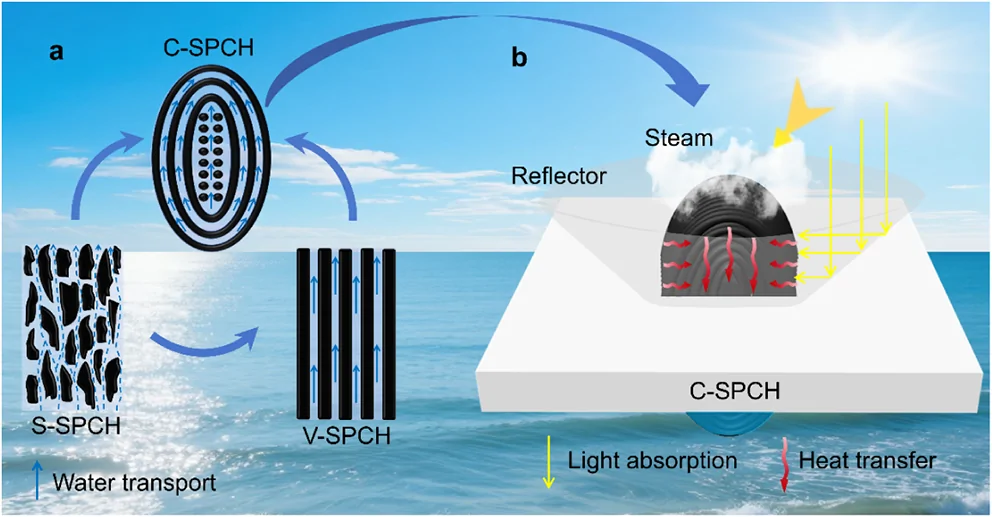
(a) Schematics of water transport in the traditional S-SPCH, V-SPCH, and the novel C-SPCH evaporators. (b) Schematic of the C-SPCH evaporator with a reflector.

## 2 Results and Discussion

To meet the stringent photothermal conversion efficiency requirements for solar-driven interfacial evaporators, C-Hair was incorporated into the polymer network of the hydrogel. Due to its excellent hydrophilicity and programmable engineering properties, PVA was selected as a representative hydrogel material.^18^ SA, a natural linear polysaccharide extracted from brown algae, was chosen to form a hybrid hydrogel with PVA. The carboxyl groups of SA can physically cross-link with Ca^2+^, rapidly forming a hydrogel. Subsequently, the hybrid hydrogel was immersed in a 5% glutaraldehyde (GA) acidic solution for 24 h. Under acidic conditions, the aldehyde groups (−CHO) of GA undergo an acetalization reaction with the hydroxyl groups (−OH) on the PVA molecular chains, leading to secondary cross-linking and the formation of a stable hydrogel.^19^ This preparation process, illustrated in Figure 2a, yielded the C-SPCH, V-SPCH, and S-SPCH evaporators (Figure 2b and Figure S1a–c).

**Figure 2:**
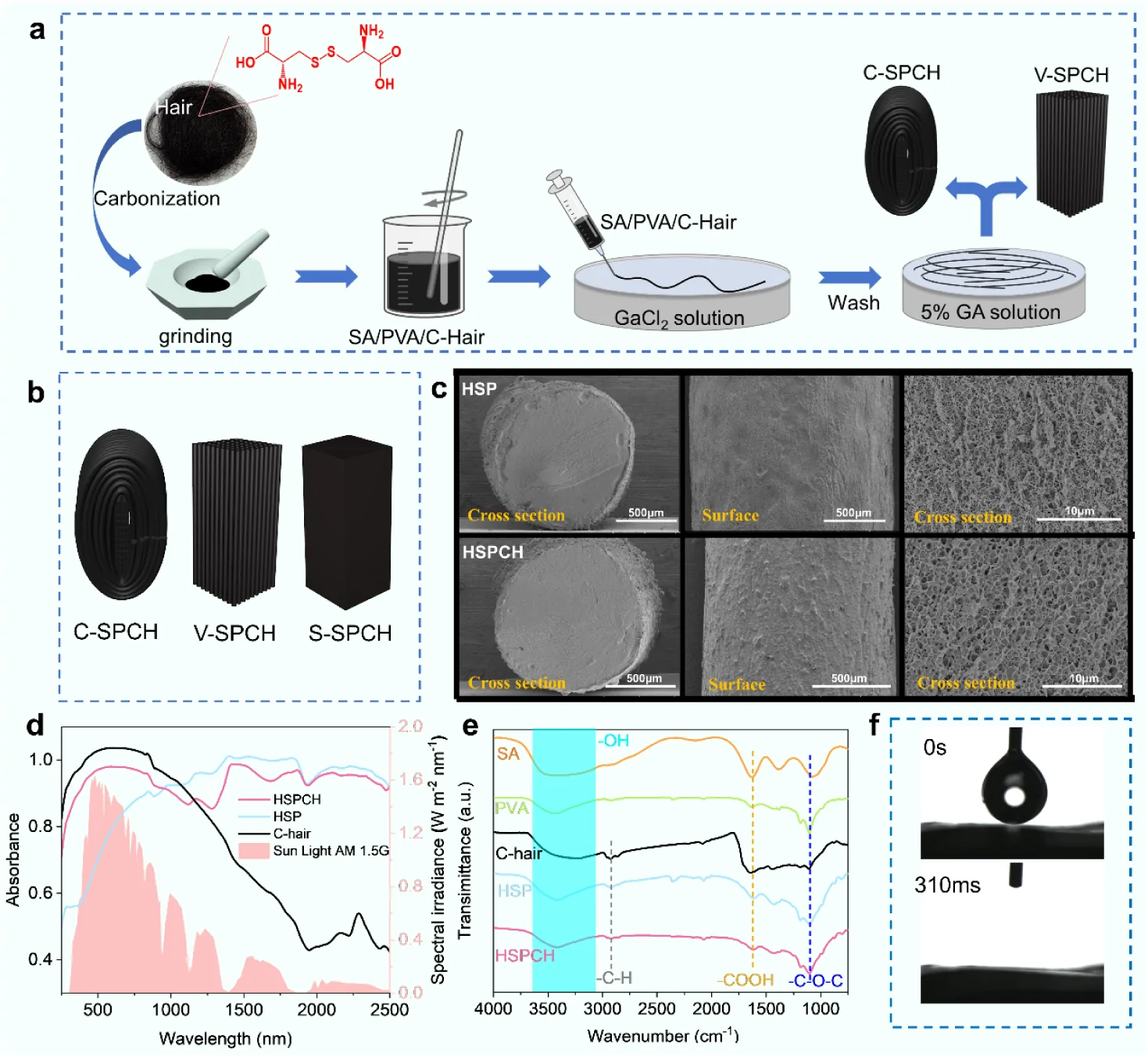
(a) The fabrication process flow of C-SPCH and V-SPCH evaporators. (b) Schematic diagrams of the C-SPCH, V-SPCH, and S-SPCH evaporators. (c) SEM images showing the surface and cross-sectional morphologies of HSP and HSPCH hydrogels. (d) UV–vis-NIR absorption spectra of C-hair, HSP, and HSPCH in the wavelength range of 250–2500 nm. (e) FT-IR spectra of SA, PVA, C-hair, HSP, and HSPCH. (f) Water contact angle measurement of the HSPCH hydrogel.

The dual PVA/SA polymer matrix is more than a simple hydrophilic blend; it is vital for both the structural stability and evaporation thermodynamics of the evaporator. Carboxyl-rich SA quickly cross-links with Ca^2+^ to solidify hydrogel tubes during wet spinning, locking in the target coiled geometry. Meanwhile, PVA forms sturdy covalent networks via glutaraldehyde acetalization, boosting mechanical strength and antiswelling performance under long-term saltwater exposure. The fragile SA-Ca^2+^ physical network is reinforced by the flexible PVA-GA covalent network. This synergy avoids structural failure during repeated swelling and dehydration, and retains the microcapillary channels needed for fast water transport and salt back-diffusion. The polar groups (−COOH and −OH) break the hydrogen-bond network of bulk water and convert free water into intermediate water. This effect greatly lowers water evaporation enthalpy and speeds up vapor generation. In short, SA shapes the coil structure and boosts hydrophilicity, while PVA offers mechanical durability. Their cooperation enables fast mass transfer, intact architecture, and strong salt resistance under long-term high-salinity working conditions.

The hydrogel synthesized from SA and PVA (HSP) is white (Figure S2), whereas the HSPCH hydrogel, obtained after incorporating C-Hair, appears black (Figure S2). Scanning electron microscopy (SEM) observations (Figure 2c) reveal that the HSP hydrogel possesses a relatively smooth surface and a cross-section characterized by densely distributed pores. In contrast, the HSPCH hydrogel exhibits a distinctly rough and uneven surface, alongside a porous cross-sectional structure. This rough surface enhances light-trapping capability, and the dense pore structure provides effective pathways for water transport. UV–vis–NIR spectroscopy further confirms the superior light absorption capability of HSPCH (Figure 2d), its average absorption is 93.5% over the wavelength range of 250–2500 nm. This is significantly higher than that of HSP (90.4%) and C-Hair (74.3%), indicating efficient light absorption across the full spectrum.

The hydrophilicity of the material is crucial for efficient water transport within the evaporator. We analyzed the functional groups of SA, PVA, C-Hair, HSP, and HSPCH using Fourier transform infrared (FTIR) spectroscopy (Figure 2e). The broad absorption peak at 3440 cm^–1^ is attributed to O–H stretching vibration, the peak at 1630 cm^–1^ corresponds to the asymmetric stretching of −COOH, the peak at 1080 cm^–1^ is associated with C–O–C group vibration, and the peak at 2922 cm^–1^ arises from C–H stretching vibration. HSPCH exhibits a strong O–H peak at 3440 cm^–1^, indicating excellent hydrophilicity, which is further supported by water contact angle measurements (Figure 2f). In summary, by incorporating carbonized hair and employing a multistep cross-linking strategy involving SA, PVA, and GA, a composite hydrogel evaporator boasting high light absorption, excellent hydrophilicity, and a porous microstructure has been successfully constructed, thus providing an ideal material platform for efficient solar-driven water evaporation.

A schematic diagram of the custom-built evaporation system is presented in Figure S3. Figure 3a displays the heating profiles of C-SPCH, V-SPCH, and S-SPCH evaporators under one-sun illumination. After 30 min, these evaporators reached surface temperatures of 43.2 °C, 42.2 °C, and 40.7 °C, respectively. As shown in Figure 3b, the temperature of the V-SPCH evaporator rises by approximately 15 to 43.2 °C after adding the reflector, underscoring the critical role the reflector plays in this process. Annular aluminum foil reflector was coaxially placed around the evaporator to redirect scattered incident light onto the coiled sidewalls while reflecting back infrared thermal emission, thereby enhancing light capture and suppressing radiative heat loss. The C-SPCH evaporator exhibited a slightly higher surface temperature than V-SPCH, which, in turn, was marginally higher than that of S-SPCH. This coil-structured design significantly enhances light-trapping capability compared to conventional bulk configurations; its uneven interface promotes improved light capture, leading to elevated temperatures. Additionally, the hydrogel demonstrates low thermal conductivity (0.3747 W·m^–1^·K^–1^), which helps reduce heat loss from the evaporator surface to the underlying water. Through photothermal evaporation tests, the C-SPCH evaporator achieved an evaporation rate of 5.36 kg·m^–2^·h^–1^, surpassing V-SPCH (4.28 kg·m^–2^·h^–1^), which similarly exceeded the rate of S-SPCH (4.03 kg·m^–2^·h^–1^), as illustrated in Figure 3c. Because the three evaporators share the same HSPCH chemical composition, this performance gap is primarily architecture-dependent. Coiling the wet-spun hydrogel filament generates continuous interfilament capillary gaps in addition to the intrinsic pores inside each filament, thereby forming a hierarchical, dual-scale water-transport network. These interconnected pathways provide rapid multidirectional water replenishment and lateral redistribution across the evaporation interface. By comparison, S-SPCH mainly relies on transport through the more tortuous pore network of a bulk hydrogel, whereas V-SPCH provides more directional transport with fewer lateral cross-connections between adjacent filaments.

**Figure 3:**
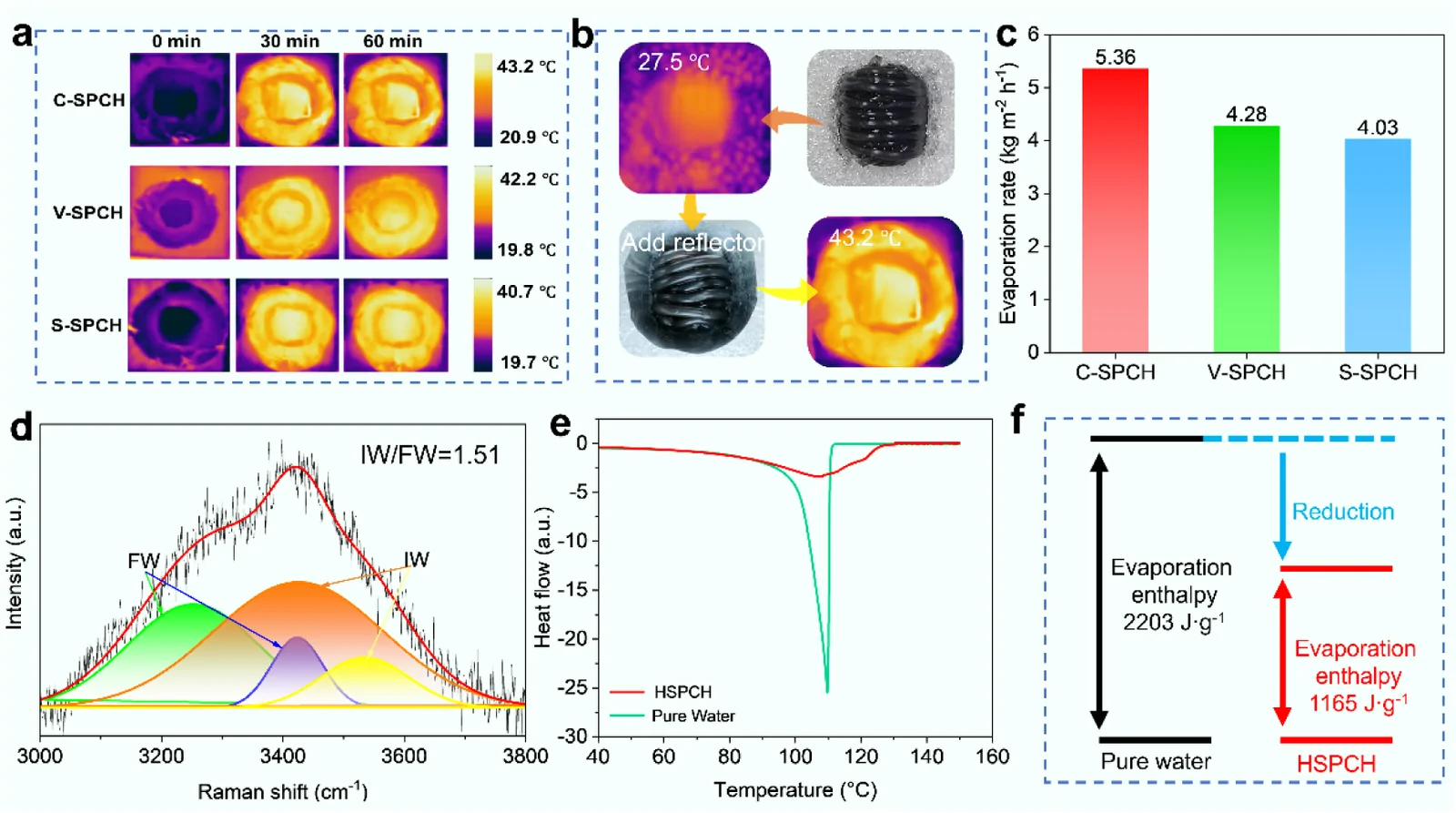
(a) Infrared thermal images showing the temperature evolution of C-SPCH, V-SPCH, and S-SPCH evaporators over 60 min under one-sun illumination. (b) Temperature change diagram of the V-SPCH evaporator before and after adding a reflector. (c) Evaporation rates of C-SPCH, V-SPCH, and S-SPCH evaporators under one-sun illumination. (d) Raman spectrum of water within the HSPCH hydrogel, illustrating peaks for free and intermediate water molecules. (e) Differential scanning calorimetry (DSC) curves of pure water and HSPCH, indicating their respective evaporation enthalpies. (f) Evaporation enthalpy of pure water and of water in HSPCH.

To elucidate the mechanism underlying the high evaporation rate, the water binding state within the HSPCH hydrogel was analyzed (Figure 3d). Gaussian fitting of the Raman spectrum of HSPCH revealed peaks at 3210 cm^–1^ and 3368 cm^–1^ attributed to free water molecules (FW), while peaks at 3425 cm^–1^ and 3619 cm^–1^ corresponded to intermediate water molecules (IW). The calculated IW/FW ratio of 1.51 indicates that the hydrogel structure disrupts the hydrogen-bonding network of bulk water, facilitating the transformation of free water into intermediate water. This transition reduces the evaporation enthalpy and enhances the evaporation rate. Furthermore, differential scanning calorimetry (DSC) was employed to determine the evaporation enthalpy of water in its pure form and within HSPCH (Figure 3e). The DSC curve of pure water exhibited a sharp and rapidly decreasing thermal flow peak, indicative of fast evaporation. In contrast, HSPCH showed a broader and more gradually declining thermal flow peak, suggesting distinct evaporation behavior compared to pure water. The measured evaporation enthalpy of water in HSPCH was 1165 J·g^–1^, significantly lower than that of pure water (2203 J·g^–1^), as illustrated in Figure 3f. This substantial reduction in evaporation enthalpy is a key factor driving the high evaporation rate observed.

The evaporation performance of three hydrogel evaporators (C-SPCH, V-SPCH, and S-SPCH) was evaluated through continuous 8-h testing in a 15 wt % NaCl solution under one-sun illumination. As shown in Figure 4a, C-SPCH exhibited the highest average evaporation rate of 5.36 kg·m^–2^·h^–1^, outperforming V-SPCH (4.28 kg·m^–2^·h^–1^) and S-SPCH (4.03 kg·m^–2^·h^–1^). Notably, C-SPCH maintained a high evaporation rate of 5.17 kg·m^–2^·h^–1^ even after 8 h.

**Figure 4:**
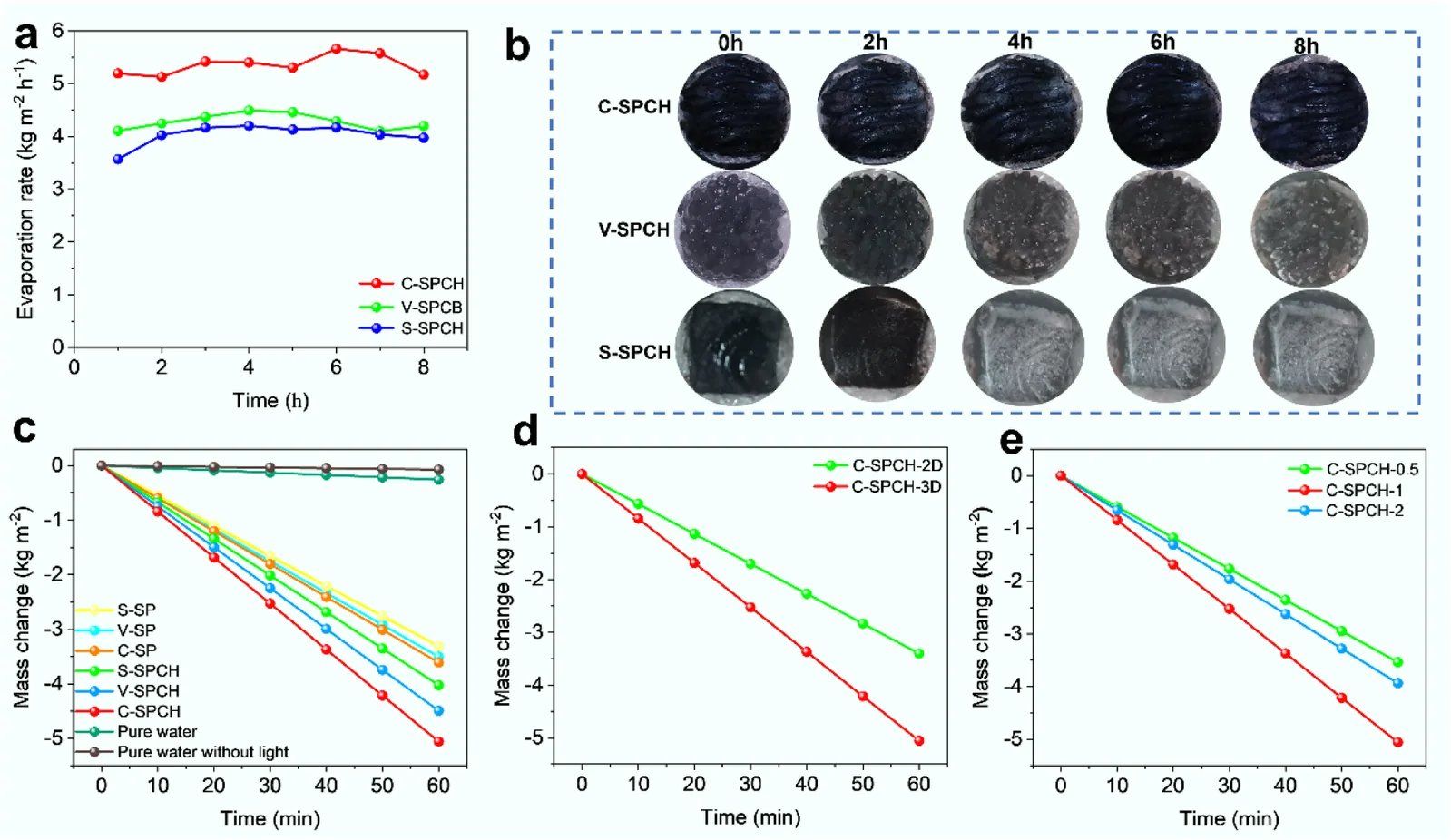
(a) Evaporation rate variations of C-SPCH, V-SPCH, and S-SPCH evaporators over 8 h in 15 wt % NaCl solution under one-sun illumination. (b) Photographic images of C-SPCH, V-SPCH, and S-SPCH evaporators during the 8 h evaporation process in 15 wt % NaCl solution under one-sun illumination. (c) Mass changes of pure water (in darkness and under illumination), S-SP, V-SP, C-SP (the abbreviations C-SP, V-SP, and S-SP denote the coiled, vertically aligned, and bulk-structured SA/PVA hydrogels, respectively, prepared without the addition of C-Hair), C-SPCH, V-SPCH, and S-SPCH after 60 min of one-sun illumination. (d) Mass changes of C-SPCH-2D (height h = 0 cm) and C-SPCH-3D (h = 0.5 cm) after 60 min of operation in 15 wt % NaCl solution under one-sun illumination. (e) Mass changes of C-SPCH-0.5 (diameter d = 0.5 mm), C-SPCH-1 (d = 1 mm), and C-SPCH-2 (d = 2 mm) after 60 min of operation in 15 wt % NaCl solution under one-sun illumination.

These differences in evaporation performance can be attributed to the surface salt crystallization behavior (Figure 4b). Throughout the experiment, no salt crystallization was observed on the surface of C-SPCH, thus maintaining a stable evaporation rate. In contrast, V-SPCH began to exhibit scattered salt particles from the fourth hour, which partially blocked water transport channels and reduced light absorption, leading to a gradual decline in its evaporation rate. S-SPCH showed noticeable crystallization as early as the second hour, with continuous salt accumulation resulting in an earlier and more pronounced decrease in performance. To further investigate salt distribution, the hydrogel samples after 8 h of illumination were freeze-dried and examined using SEM (Figure S4). The results indicated that although salt particles were present on both the cross sections and surfaces of the hydrogels, their internal pore structures were not completely blocked. This observation provides a microscopic explanation for C-SPCH’s ability to sustain stable evaporation.

As a control, pure water without any evaporator showed negligible mass loss (0.26 kg·m^–2^) after 60 min of illumination, attributable to the absence of both a light-absorbing material and a solar-driven interfacial evaporation structure. Furthermore, hydrogel evaporators fabricated without C-Hair as the light-absorbing material (C-SP, V-SP, S-SP) also exhibited relatively low water evaporation amounts (3.61 kg·m^–2^, 3.5 kg·m^–2^, and 3.31 kg·m^–2^, respectively). In comparison, the incorporation of C-Hair significantly enhanced the evaporation performance (C-SPCH: 5.06 kg·m^–2^, V-SPCH: 4.5 kg·m^–2^, S-SPCH: 4.03 kg·m^–2^), as summarized in Figure 4c, thereby demonstrating the critical role of C-Hair in improving evaporation efficiency.

The influence of evaporator height on the evaporation rate was also investigated (Figure 4d). After 60 min of one-sun irradiation, the two-dimensional evaporator C-SPCH-2D (height h = 0 cm) achieved an evaporation amount of 3.404 kg·m^–2^, whereas the three-dimensional evaporator C-SPCH-3D (h = 0.5 cm) yielded a significantly higher value of 5.51 kg·m^–2^. This enhancement is primarily attributed to the sidewall cooling evaporation effect in the 3D structure, which maximizes energy reuse and effectively reduces heat loss from the photothermal layer. Furthermore, the effect of hydrogel diameter on the evaporation rate was studied (Figure 4e). The evaporation rates for C-SPCH-0.5 (diameter d = 0.5 mm), C-SPCH-1 (d = 1 mm), and C-SPCH-2 (d = 2 mm) were 3.54 kg·m^–2^·h^–1^, 5.06 kg·m^–2^·h^–1^, and 3.94 kg·m^–2^·h^–1^, respectively. Among these, the C-SPCH sample with a diameter of 1 mm demonstrated optimal evaporation performance, suggesting the existence of an ideal diameter for efficient evaporation, as both excessively large and small diameters proved unfavorable.

To investigate the impact of varying solar light intensities on the C-SPCH evaporator’s evaporation performance, experiments were conducted under multiple light conditions. Under 3-sun illumination, the evaporation rate achieved 11.15 kg·m^–2^·h^–1^ (Figure 5a). Concurrently, the evaporator’s surface temperatures were monitored under different light intensities (Figure S5). After 1 h of illumination, the surface temperature reached 43.2 °C under 1-sun, increased to 50.7 °C under 2-sun, and further rose to 62.1 °C under 3-sun conditions. These findings indicate that increased light intensity significantly elevates the evaporator’s surface temperature, thereby effectively enhancing the evaporation process.

**Figure 5:**
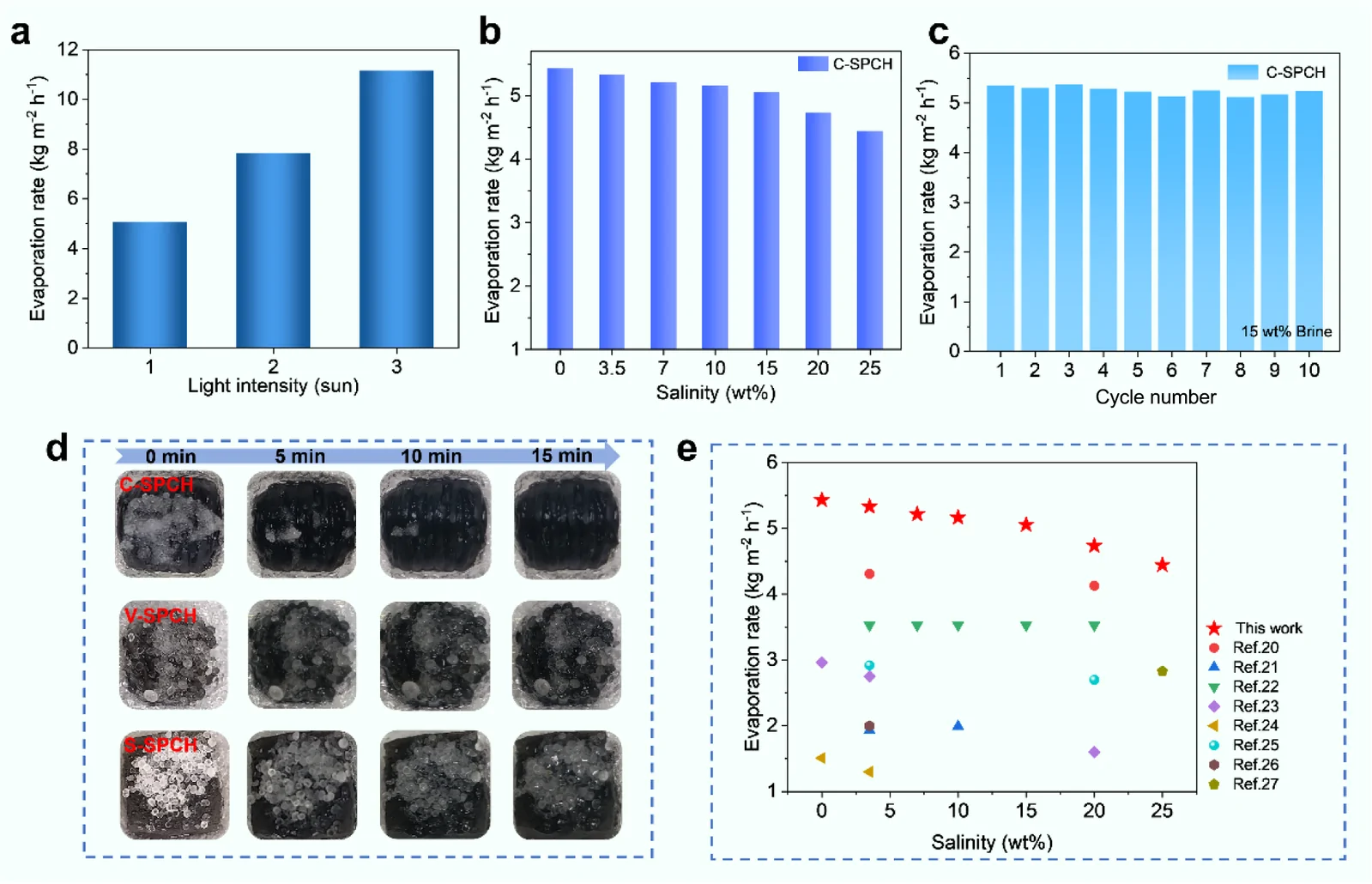
(a) Evaporation rates of the C-SPCH evaporator under varying light intensities. (b) Evaporation rates of C-SPCH in NaCl solutions with concentrations ranging from 0 to 25 wt %. (c) Evaporation rates of C-SPCH during eight consecutive days of cycling tests in 15 wt % NaCl solution under one-sun illumination. (d) Schematic illustration of the back-diffusion of 0.1 g NaCl on the surfaces of C-SPCH, V-SPCH, and S-SPCH evaporators. (e) Comparison of evaporation rates between C-SPCH and various other hydrogel evaporators across different salinity levels.

The influence of salt concentration on evaporation performance was also evaluated. As depicted in Figure 5b, the evaporation rate gradually decreased with increasing salt concentration. Nonetheless, even in a near-saturated brine environment (25 wt % NaCl), the C-SPCH evaporator maintained a robust evaporation rate of 4.45 kg·m^–2^·h^–1^, underscoring its reliable performance under high-salinity conditions. Long-term stability tests further corroborated this reliability: during a 10-day continuous operation (with 8 h of illumination per day) in 15 wt % NaCl solution, the evaporation rate remained stable (Figure 5c), demonstrating the C-SPCH evaporator’s excellent long-term durability.

To elucidate its salt resistance mechanism, 0.1 g of NaCl particles were placed on the photothermal layer to investigate the salt back-diffusion capability (Figure 5d). The C-SPCH rapidly wetted and completely dissolved the surface salt particles within 10 min, whereas visible salt residues persisted on both V-SPCH and S-SPCH even after 15 min. This observation confirms C-SPCH’s outstanding salt ion transport capacity, which facilitates the timely back-diffusion of crystallized salt from the surface into the bulk water. This also accounts for the absence of surface salt crystallization after 8 h of continuous illumination (Figure 4b). Significantly, across various salinity conditions, the evaporation rate of C-SPCH markedly outperformed other hydrogel evaporators (Figure 5e),^20–27^ underscoring its superior overall performance. Figure S6 further illustrates the efficient water transport channels within the evaporator, which not only facilitate rapid water delivery to the surface but also simultaneously enable the transport of salt particles back into the bulk water. In summary, the C-SPCH evaporator exhibits high evaporation efficiency, positively correlating with light intensity, maintains stable and excellent evaporation performance in high-salinity environments, and demonstrates exceptional salt back-diffusion capability and long-term operational stability.

The prominent high-salinity performance of C-SPCH can therefore be understood as a dynamic balance between salt accumulation at the evaporating interface and salt removal through the hydrated transport network. During evaporation, dissolved Na^+^ and Cl^–^ are carried toward the surface with the replenishing water and become locally concentrated as water is continuously removed. In C-SPCH, however, the interfilament capillary gaps generated by the coil geometry, together with the interconnected pores inside each hydrogel filament, maintain continuous water influx and provide multiple pathways for ionic diffusion back toward the bulk solution. Rapid liquid exchange dilutes the near-surface brine and sustains a concentration gradient that drives back-diffusion, thereby delaying local supersaturation, heterogeneous nucleation, and growth of a continuous salt crust. If incipient crystals form, persistent rewetting promotes their redissolution before extensive surface blockage occurs. In contrast, the less interconnected transport pathways in V-SPCH and especially S-SPCH are more susceptible to concentration polarization and local channel blockage, consistent with the earlier appearance of visible surface crystals in Figure 4b. The complete disappearance of 0.1 g of deposited NaCl on C-SPCH within 10 min (Figure 5d), the absence of visible crystallization after 8 h in 15 wt % brine (Figure 4b), and the preservation of internal pore space after testing (Figure S4) collectively support this coupled water-replenishment/ion-back-diffusion mechanism.

Figure 6a illustrates the outdoor experimental setup employed to evaluate the C-SPCH evaporator’s practical seawater desalination performance using natural seawater. The seawater was collected from the Xiasha Beach Resort in Changle, Fuzhou (25.8°N, 119.6°E). The outdoor environmental temperature on that day was between 28 and 37 °C, and the relative humidity was 46%. Figure 6b displays the solar intensity, ambient temperature variations, and cumulative freshwater collection measured in Fuzhou, Fujian Province, from 09:00 to 17:00 on August 8, 2025. The solar intensity escalated from 760 W·m^–2^ at 09:00 to a maximum of 920 W·m^–2^ at noon, subsequently decreasing to 420 W·m^–2^ by 16:00. The hourly water collection trend closely mirrored solar irradiance variations, thus confirming light intensity as the primary driving factor for evaporation. Notably, no salt crystallization was observed on the C-SPCH surface throughout the testing period, attesting to its excellent salt rejection capability. Inductively coupled plasma optical emission spectrometry (ICP-OES) analysis of both seawater and collected freshwater revealed significantly reduced ion concentrations in the treated water, falling well below the drinking water standards established by the World Health Organization (WHO) (Figure 6c). This unequivocally confirms the evaporator’s effective desalination performance.

**Figure 6:**
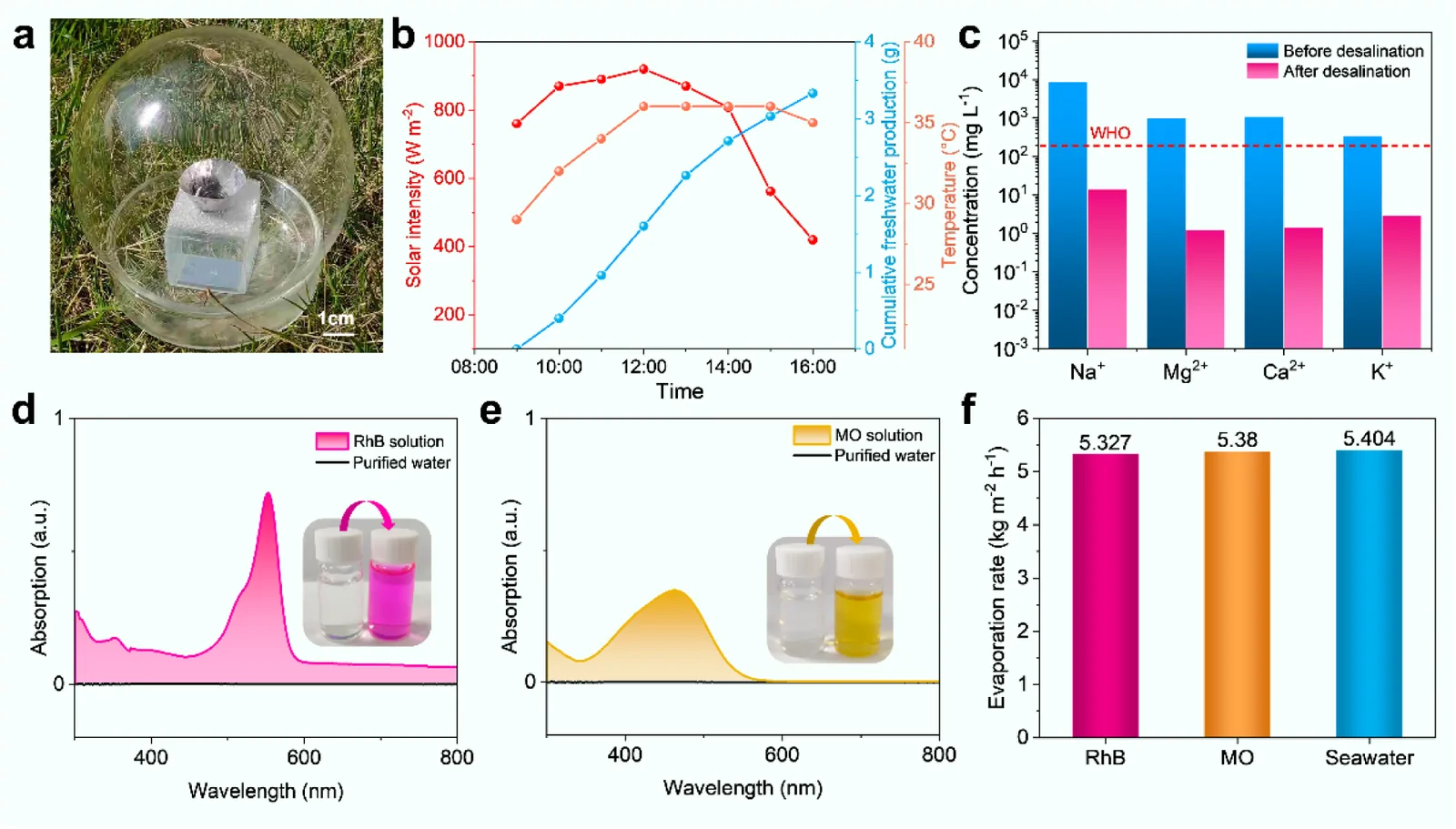
(a) Schematic diagram of the C-SPCH evaporator installed in an outdoor experimental setup. (b) Temporal variations in system mass change, solar irradiance, and temperature during outdoor operation. (c) Changes in metal ion concentrations before and after seawater desalination. (d, e) UV–vis spectra of RhB (d) and MO (e) solutions (20 mg/L) before and after C-SPCH treatment. Insets show corresponding photographic images of the solutions. (f) Evaporation rates of the C-SPCH evaporator in RhB solution (20 mg/L), MO solution (20 mg/L), and real seawater.

The C-SPCH evaporator’s purification capability for organic dye-contaminated water was also thoroughly verified. As depicted in Figure 6d and 6e, the characteristic absorption peaks of Rhodamine B and methyl orange vanished in the treated water, signifying effective removal of these representative organic dyes. Under one-sun illumination, the C-SPCH exhibited consistent high evaporation rates across different water systems (Figure 6f): 5.33 kg·m^–2^·h^–1^ in 20 mg·L^–1^ Rhodamine B solution, 5.38 kg·m^–2^·h^–1^ in 20 mg·L^–1^ methyl orange solution, and 5.4 kg·m^–2^·h^–1^ in natural seawater. These results unequivocally demonstrate C-SPCH’s sustained high evaporation performance across various complex water qualities. Moreover, as Figure S7 illustrates, the evaporation rate decreased only slightly with increasing methyl orange concentration while still maintaining a high level, further confirming the evaporator’s robust adaptability and stability under varying pollutant concentrations. In conclusion, the C-SPCH evaporator showcases excellent freshwater collection capacity, effective desalination, and efficient organic dye removal in practical outdoor environments. Its stable and high evaporation performance under diverse water quality conditions highlights its significant potential for applications in both seawater desalination and wastewater purification.

The superior performance of the C-SPCH evaporator originates from a four-tier synergistic hierarchy. At the optical level, carbonized hair ensures broadband absorption (93.5%), while the coiled surface topography promotes multireflection light trapping. Thermally, low thermal conductivity (0.3747 W·m^–1^·K^–1^) minimizes heat loss, and the three-dimensional sidewall cooling effect enables environmental energy harvesting. Regarding water activation, abundant polar groups (−COOH and −OH) disrupt hydrogen bonding, shifting free water to intermediate water and reducing evaporation enthalpy from 2203 J·g^–1^ to 1165 J·g^–1^. For mass transport, capillary channels and interconnected pores enable rapid water supply and simultaneous salt back-diffusion, preventing crystallization even in 25 wt % brine. These four levels mutually reinforce one another, collectively delivering both ultrahigh evaporation rate (5.36 kg·m^–2^·h^–1^) and exceptional long-term stability.

## 3 Conclusion

In summary, this study successfully developed a composite hydrogel solar evaporator (C-SPCH) with a novel coil-structured design. During simulated seawater desalination, the evaporator reached an evaporation rate of 5.404 kg·m^–2^·h^–1^, and the condensed freshwater meets WHO drinking water standards, confirming its viability for offshore freshwater harvesting. For hypersaline wastewater (0–25 wt % NaCl), the device relies on directional salt back-diffusion and self-cleaning effects to drastically mitigate surface salt crystallization, retaining stable performance over 20 h of continuous operation. This finding presents an innovative route to resolve the critical issue of salt concentration, crystal deposition and channel clogging in industrial hypersaline wastewater remediation. It is worth noting that all evaluations are limited to laboratory simulated setups. Further field-scale investigations are required to confirm its long-term durability and tolerance to real-world conditions such as unstable sunlight and multi-ion composite polluted water. Furthermore, the system exhibits reliable and highly efficient evaporation capabilities under various water quality conditions, suggesting promising application potential in seawater desalination and high-salinity wastewater treatment. The superior performance of C-SPCH originates from its multiscale structural design and material synergy. Specifically, the microcapillary channels formed by the coil-like arrangement, coupled with the material’s internal pore structure, significantly enhance water transport and salt diffusion. The surface microstructure effectively improves light-trapping efficiency, while the hydrophilic groups within the hydrogel contribute to reducing the evaporation enthalpy of water molecules. These factors collectively promote efficient and stable solar-thermal-evaporation energy conversion. Through structural innovation and material hybridization, this study offers novel insights for the design of high-performance solar interfacial evaporation systems, thereby contributing to the advancement of sustainable seawater desalination and high-salinity wastewater treatment technologies.

## Supplementary Material



## Notes

Details on chemicals and materials, sample preparation, characterizations, supplementary note on calculations, and characterization and analysis (PDF)
