# Supplementary material for: Bioinspired Coiled Composite Hydrogel Evaporator with Carbonized Hair for Efficient Solar Desalination and Salt Rejection
Source: ACS Omega. 2026 Aug 18;11(34):52114–22. doi: 10.1021/acsomega.6c07905 (PMC13625089; doi:10.1021/acsomega.6c07905)
Supplement: Supplementary file 1 [file ao6c07905_si_001.pdf]

Supporting Information

Bioinspired Coiled Composite Hydrogel Evaporator with Carbonized Hair for Efficient Solar Desalination and Salt Rejection

Yi Liu,[a, 1] Liqiang Zhang,[a, 1] Haoqi Ni,[a, 1] Shuozhen Chen,[a] Ran Li,[a] Jiaxian Zheng,[a,b] * Xiangfeng Lin,[a,] * Weiwei Zhao,[c,]* and Zhanhui Yuan[a,] *

[a]College of Materials Engineering, Fujian Agriculture and Forestry University, Fuzhou 350108, P.R. China

[b]State Key Laboratory of Advanced Papermaking and Paper-based Materials , South China University of Technology, Guangzhou 510640, P.R. China

[c]Birmingham Centre for Energy Storage & School of Chemical Engineering, University of Birmingham, Edgbaston, B15 2TT, United Kingdom

[1]These authors contributed equally to this work.

E-mail: zjxcms@fafu.edu.cn; xiangfenglin@fafu.edu.cn; w.zhao@bham.ac.uk; zhanhuiyuan@fafu.edu.cn

CHEMICALS AND MATERIALS

Rhodamine B, polyvinyl alcohol, and sodium alginate were purchased from Shanghai Aladdin Bio-Chem Technology Co., Ltd. Insulating foam was obtained from a local market in Fuzhou. Sodium chloride, calcium chloride, glutaraldehyde, and methyl orange were purchased from Sinopharm Chemical Reagent Co., Ltd.

SAMPLE PREPARATION

HSPCH was fabricated from an aqueous mixture containing 3 wt% SA, 1 wt% PVA, and carbonized human hair (C-Hair), whereas HSP was prepared from a solution of 3 wt% SA and 3 wt% PVA. The preparation process is illustrated in Figure 2a. The precursor solution was injected into a coagulation bath containing a $CaCl_2$ solution, where it was allowed to soak for 12 hours to form tubular hydrogels. Subsequently, the hydrogels were immersed in a 5% glutaraldehyde (GA) solution for 24 hours to induce secondary cross-linking via acetal polycondensation between PVA and GA. Afterward, the samples were thoroughly rinsed with ultrapure water to obtain stable hydrogel tubes.

These hydrogel tubes were then cut and arranged into two configurations: a coiled solar evaporator (C-SPCH) and a vertically aligned evaporator (V-SPCH). For comparison, a bulk-structured hydrogel evaporator (S-SPCH) was prepared using a bulk mold. To enhance the evaporation performance, a reflective foil was attached to the external surfaces of the hydrogel evaporators. Additionally, control samples without C-Hair were prepared under identical conditions, resulting in coiled (C-SP), vertically aligned (V-SP), and bulk-structured (S-SP) hydrogel evaporators.

The coiled C-SPCH evaporator was fabricated via a manual self-rolling approach. Specifically, the as-spun HSPCH hydrogel tube, with an outer diameter of 1.0 mm, was naturally coiled by hand winding it into a compact spiral configuration without the aid of external templates or spacers. Adjacent turns were maintained in close contact with each other, relying on the intrinsic viscoelasticity and surface tackiness of the wet hydrogel to preserve the coiled morphology. After ambient drying, the C-SPCH

evaporator with a tightly packed coiled geometry was obtained, featuring a fiber diameter of 1.0 mm and a coil inner diameter of approximately 15 mm. It should be noted that the close packing of adjacent fibers does not obstruct liquid flow; rather, the inter-fiber gaps at the microscale, combined with the intrinsic microporous structure of the hydrogel, collectively function as capillary channels that facilitate rapid water transport and salt back-diffusion. For comparison, vertically aligned (V-SPCH) and bulk (S-SPCH) samples were prepared using identical hydrogel compositions but without the coiling treatment.

To evaluate the photothermal water evaporation performance, a xenon lamp was used as the light source to simulate solar irradiation. The evaporation rates of the hydrogel evaporators were measured under one-sun illumination (1 $kW \cdot m^{-2}$). The ambient temperature and humidity were maintained between 23-25°C and 40-45%, respectively.

CHARACTERIZATIONS

Scanning electron microscopy (SEM, Hitachi SU8010) and UV-Vis-NIR spectrophotometry (Lambda 750S) were employed to characterize the morphology of HSP and HSPCH and to investigate their light absorption capabilities. The dynamic water contact angle on the sample surface was measured using a contact angle meter (DSA 30, 0-180°). The chemical composition of the samples was determined using Fourier transform infrared (FTIR) spectroscopy (Nicolet 7000). Evaporation performance was evaluated under simulated sunlight provided by a xenon lamp (CEL-HXF300). The evaporation rate (v, $kg \cdot m^{-2} \cdot h^{-1}$) and evaporation efficiency (η, %) were determined by continuously monitoring mass changes using a precision electronic balance. The dye removal performance of the evaporator was assessed using a UV spectrophotometer (UV-2600). The surface temperature of the solar evaporator was recorded using an infrared camera (FLIR ONE PRO). Elemental concentrations within the samples were analyzed using an inductively coupled plasma optical emission spectrometer (ICP-OES, Agilent 5110) for precise determination of elemental composition. Raman spectroscopy (Horiba LabRAM HR Evolution) was employed to

analyze the different water molecular states within the hydrogel. This comprehensive set of measurements offers valuable data for understanding the photothermal properties and potential applications of the hydrogel as a sustainable material. Differential scanning calorimetry (DSC, HITACHI DSC200) measurements involved heating the samples from an initial temperature of 20 °C to a final temperature of 170 °C at a linear heating rate of 5 °C/min. This method enables a detailed analysis of the thermal properties and phase transitions of water within the hydrogel and also provides insights into how the evaporation enthalpy changes under controlled conditions.

SUPPLEMENTARY NOTE ON CALCULATIONS

Calculation of the evaporation rate:

$$m = \Delta m/(At)$$

where m ($kg\ m^{-2}\ h^{-1}$) is the evaporation rate, Δm (kg) is the mass losses of liquids, A is the light radiation area of evaporators, and t is the evaporation time.

The evaporation efficiencies can be calculated using the following equation:

$$\eta = \frac{m \times E_{equ}}{P_0}$$

where m is the evaporation rate, E_{equ} is the equivalent evaporation enthalpy of water, and P_0 is the solar irradiation power of one sun (1 kW m^{-2}).

The E_{equ} of water in C-SPCH can be estimated by vaporizing the water with identical power input (U_{in}), which can be represented as:

$$U_{in} = E_0 m_0 = E_{equ} m$$

where E_0 is the evaporation enthalpy of water (2450 J g^{-1}), m_0 is a mass change of bulk water (without a hydrogel evaporator) under dark conditions, and mg is the mass change of the hydrogels under the same conditions

CHARACTERIZATION AND ANALYSIS

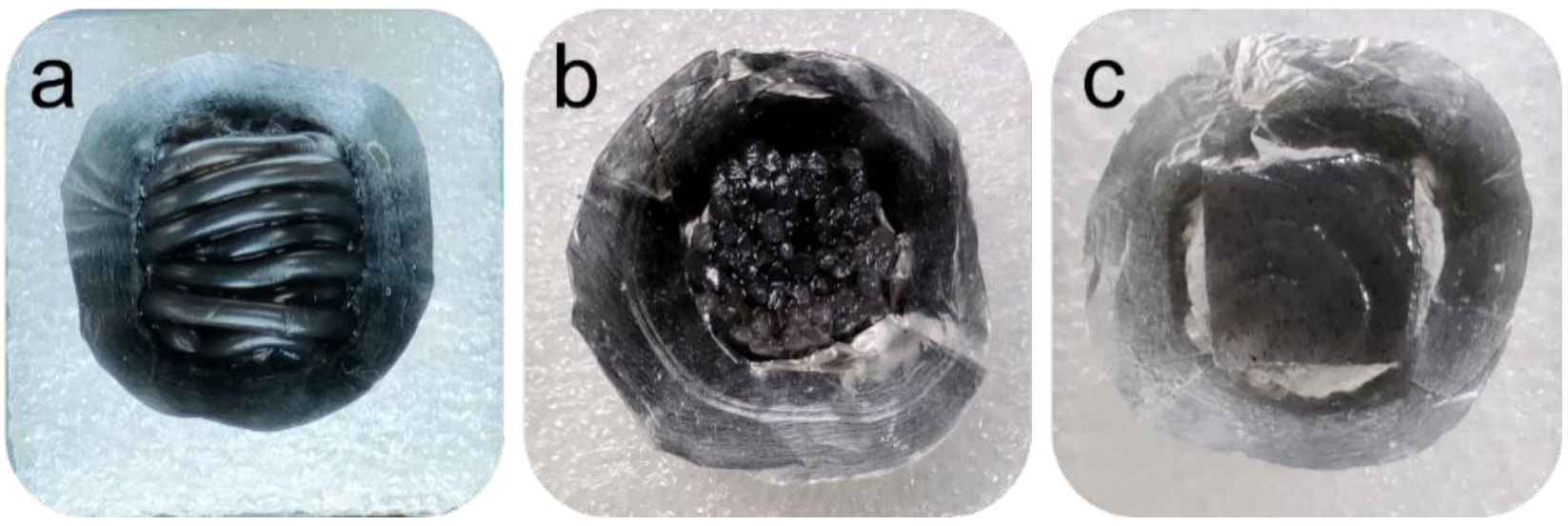

Fig. S1. Schematic diagrams of the a) C-SPCH, b) V-SPCH, c) S-SPCH evaporators.

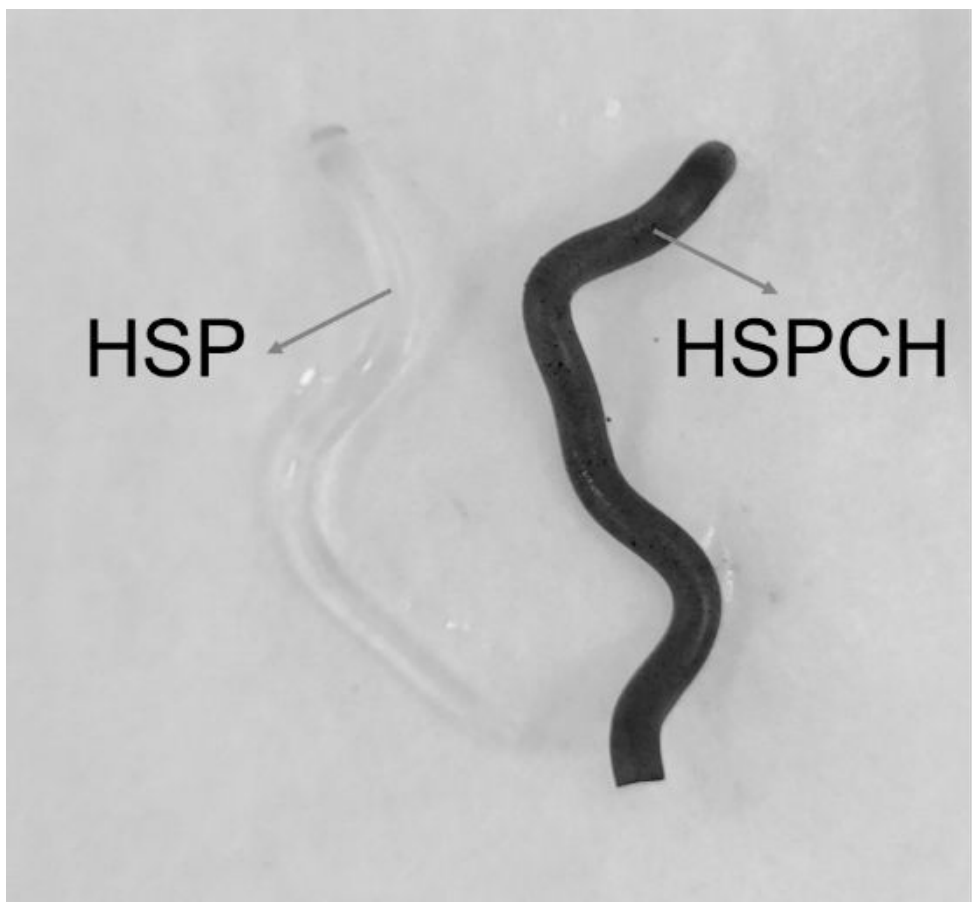

Fig. S2. Images of HSP and HSPCH hydrogels.

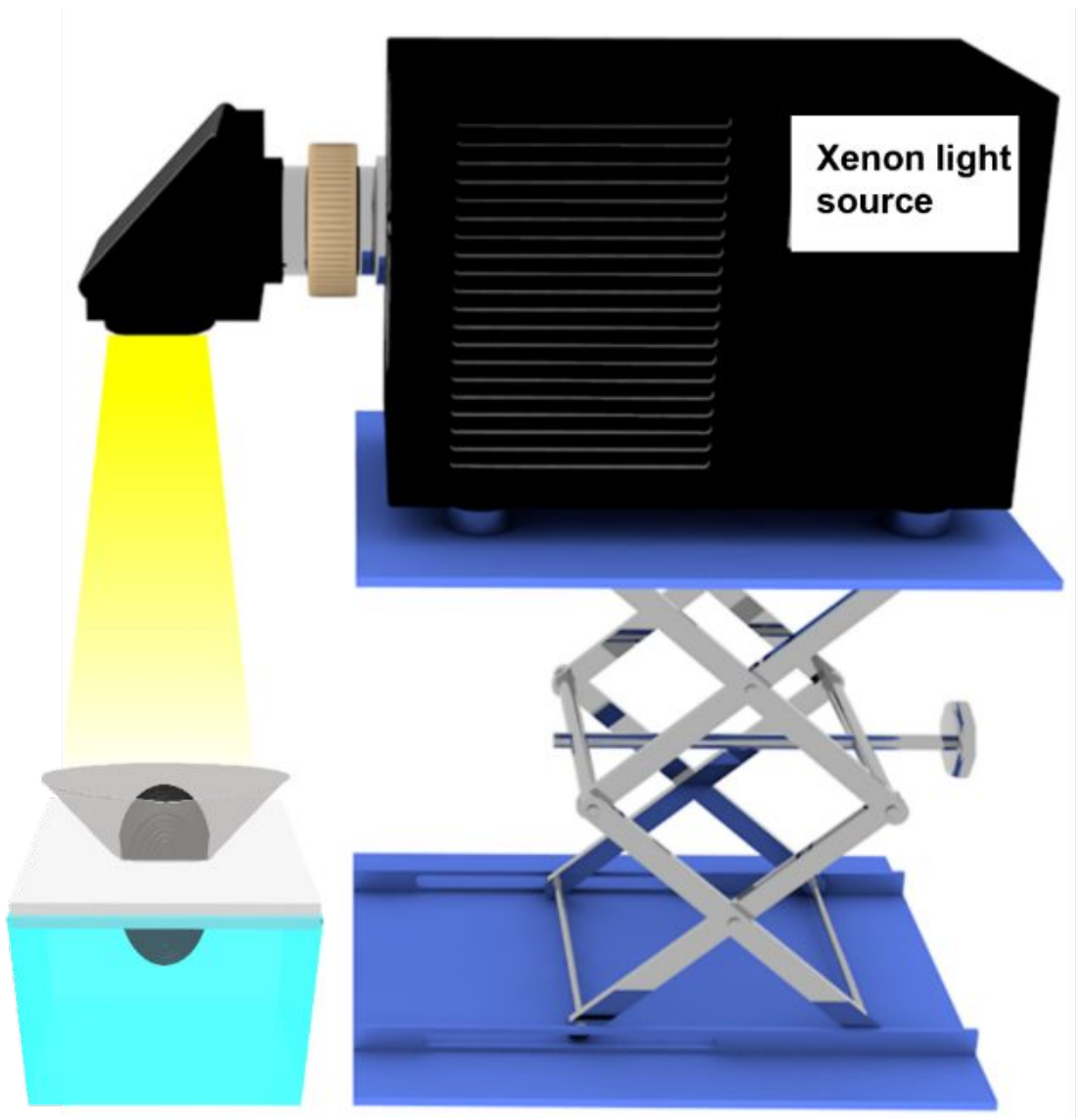

Fig. S3. Schematic diagram of the indoor evaporation system.

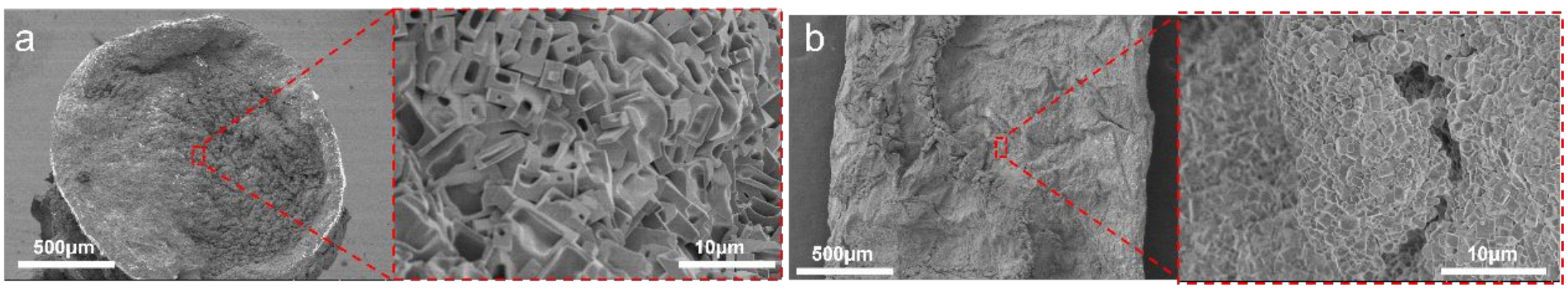

Fig. S4. Cross-sectional (a) and surface (b) SEM images of the hydrogel after 8 h of light-driven evaporation in 15 wt% brine under 1 sun illumination.

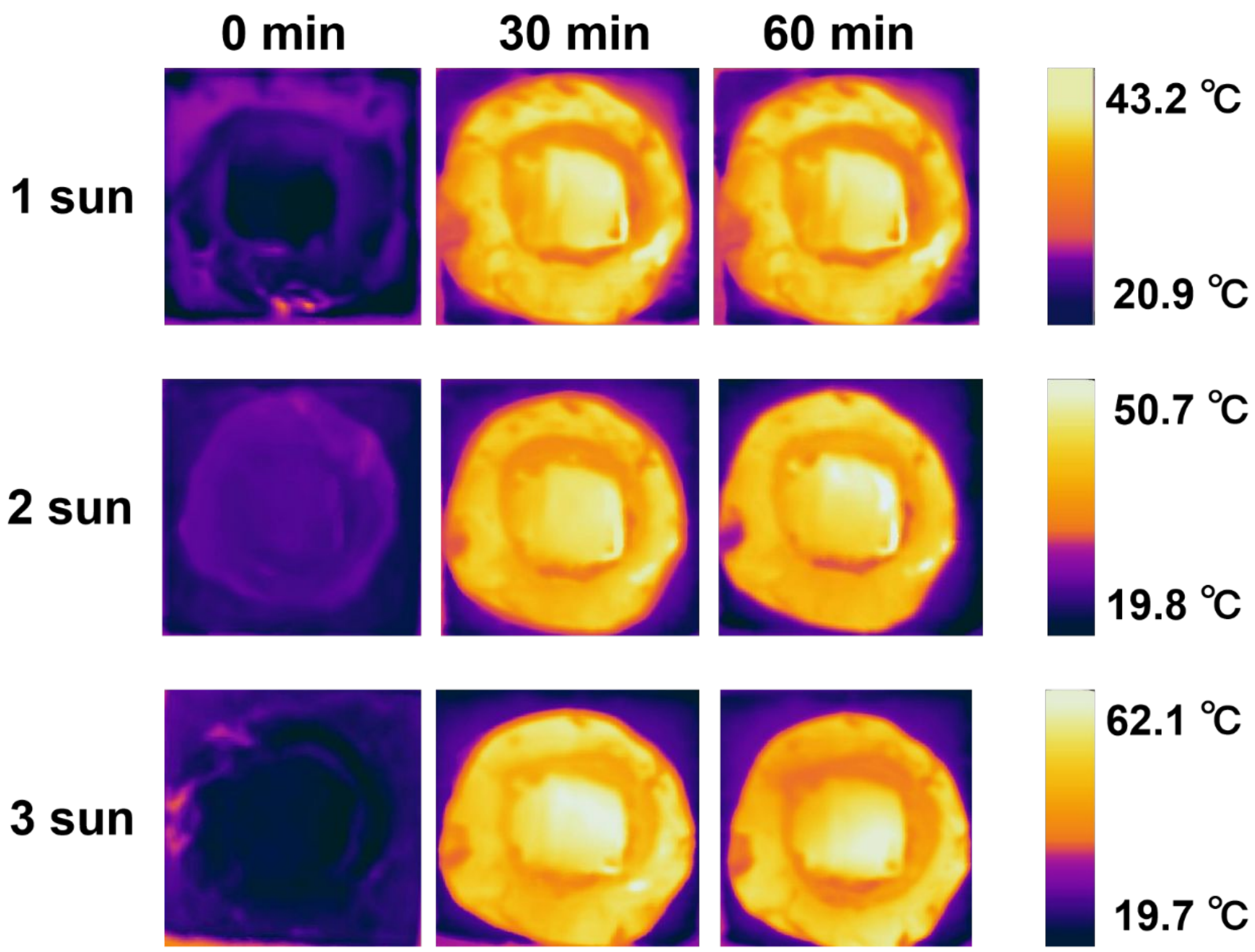

Fig. S5. Surface temperature variations of the C-SPCH evaporator under different light intensities.

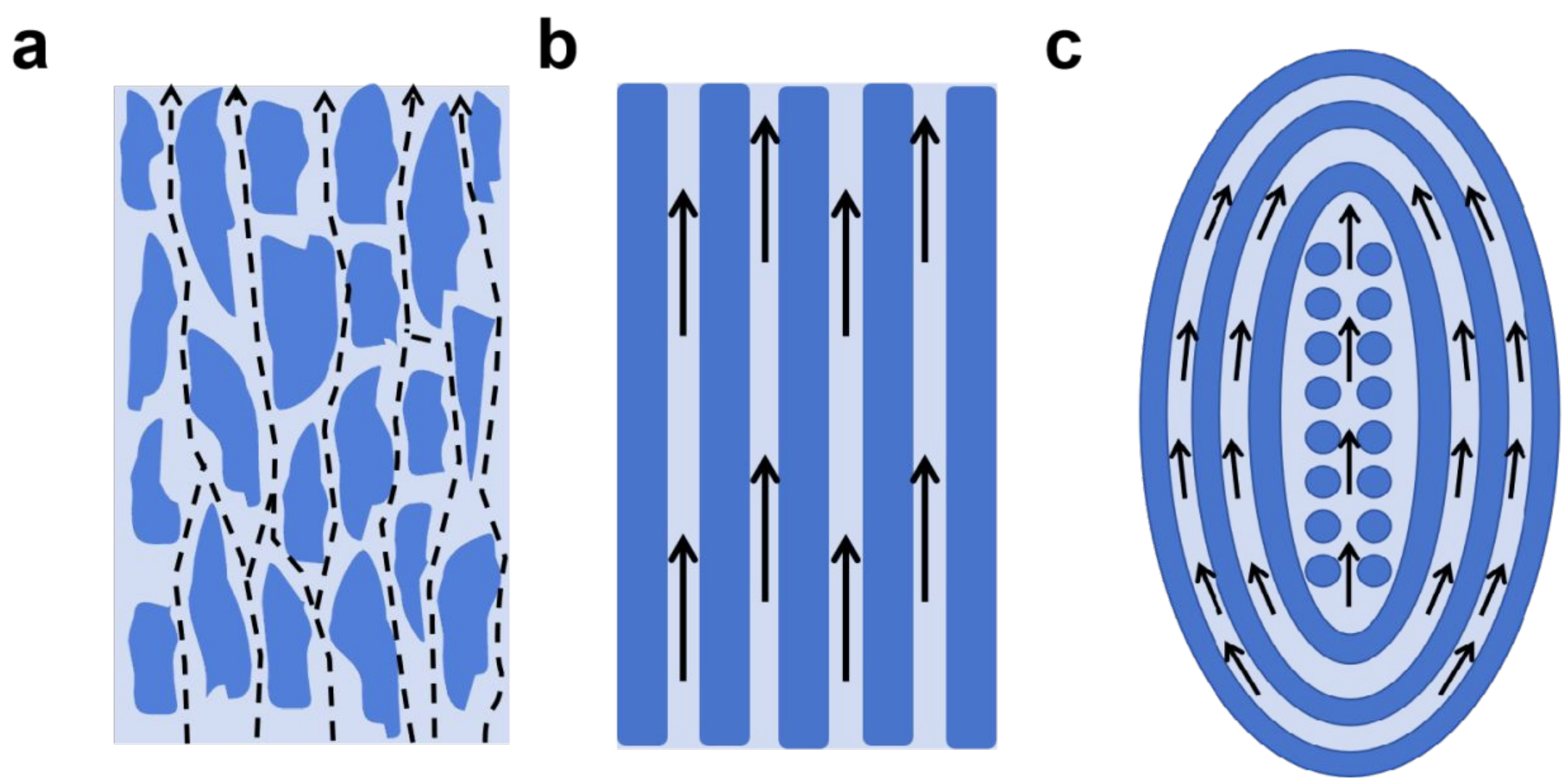

Fig. S6. Water transport channels of the (a) S-SPCH, (b) V-SPCH, (c) C-SPCH evaporators.

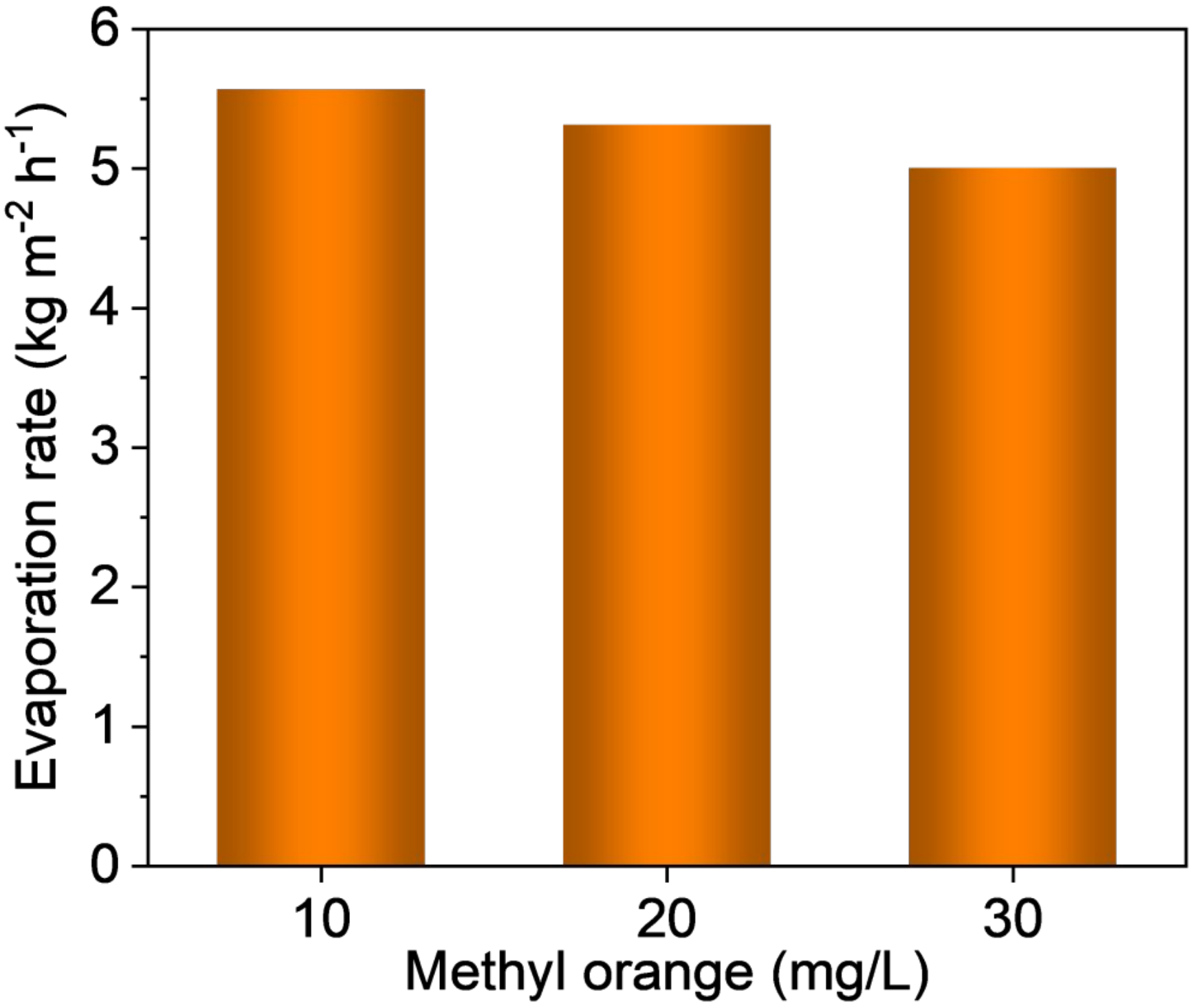

Fig. S7. Evaporation rates of the C-SPCH evaporator in different concentrations of MO solution.
